# Modulation of the oxidative plasmatic state in gastroesophageal reflux disease with the addition of rich water molecular hydrogen: A new biological vision

**DOI:** 10.1111/jcmm.13569

**Published:** 2018-03-07

**Authors:** Sara Franceschelli, Daniela Maria Pia Gatta, Mirko Pesce, Alessio Ferrone, Giuseppe Di Martino, Marta Di Nicola, Maria Anna De Lutiis, Ester Vitacolonna, Antonia Patruno, Alfredo Grilli, Mario Felaco, Lorenza Speranza

**Affiliations:** ^1^ Department of Medicine and Science of Aging University “G. D' Annunzio” Chieti Italy; ^2^ Department of Psychological, Health and Territorial Sciences University “G. D' Annunzio” Chieti Italy; ^3^ Laboratory of Biostatistics Department of Medical, Oral and Biotechnological Sciences University “G. D' Annunzio” Chieti Italy

**Keywords:** electrolysed reduced water, gastroesophageal reflux disease, molecular hydrogen, nitric oxide, oxidative stress, proton pump inhibitors

## Abstract

Gastroesophageal reflux disease (GERD), a clinical condition characterized by reflux of gastroduodenal contents in the oesophagus, has proved to demonstrate a strong link between oxidative stress and the development of GERD. Proton pump inhibitors (PPIs) have been universally accepted as first‐line therapy for management of GERD. The potential benefits of electrolysed reduced water (ERW), rich in molecular hydrogen, in improving symptoms and systemic oxidative stress associated with GERD was assessed. The study was performed on 84 GERD patients undergoing control treatment (PPI + tap water) or experimental treatment (PPI + ERW) for 3 months. These patients were subjected to the GERD‐Health Related Quality of Life Questionnaire as well as derivatives reactive oxigen metabolites (d‐ROMs) test, biological antioxidant potential (BAP) test, superoxide anion, nitric oxide and malondialdehyde assays, which were all performed as a proxy for the oxidative/nitrosative stress and the antioxidant potential status. Spearman's correlation coefficient was used to evaluate the correlation between scores and laboratory parameters. Overall results demonstrated that an optimal oxidative balance can be restored and GERD symptoms can be reduced rapidly via the integration of ERW in GERD patients. The relative variation of heartburn and regurgitation score was significantly correlated with laboratory parameters. Thus, in the selected patients, combination treatment with PPI and ERW improves the cellular redox state leading to the improvement of the quality of life as demonstrated by the correlation analysis between laboratory parameters and GERD symptoms.

## 1 INTRODUCTION

Generally, oxidative stress can be easily defined as the condition arising from the imbalance between toxic reactive oxygen species (ROS) and the antioxidant systems.^1^ As the first step in generating persistent ROS, the majority of superoxide anion radicals (${}^{\cdot }O{{}_{2}}^{-}$) are generated in mitochondria by electron leakage from the electron transport chain. Superoxide dismutase converts the superoxide anion to hydrogen peroxide (H_2_O_2_), which is metabolized by glutathione peroxidase and catalase to generate water. Highly reactive hydroxyl radicals (${}^{\cdot }\text{OH}$) are generated from H_2_O_2_ via the Fenton or Weiss reaction in the presence of catalytically active metals, such as Fe^2+^ and Cu^2+^.^2^ In the last few years, molecular hydrogen (H_2_) has been pointed out to be a preventive and therapeutic antioxidant. Several research articles have confirmed the efficacy of H_2_ both in vitro than in various animal models.^3^ H_2_, because of its physicochemical properties of solubility, neutrality and small size, has some high distribution properties allowing it to quickly penetrate bio‐membranes and get to intracellular compartments, where it can carry out its biological effects. Ohsawa et al^4^ first reported that pre‐treatment with H_2_ inhalation ameliorates brain lesions after cerebral infarction in rats. Emerging data have shown that H_2_‐rich water has beneficial effects on oxidative stress‐related diseases such as cancer, arteriosclerosis, diabetes, neurodegenerative diseases and the side effects of haemodialysis.^5^ Moreover, it was reported that H_2_ treatment resulted in significantly improved gastrointestinal (GI) transit, protected organs from tissue damage induced by ischaemia reperfusion and effectively ameliorated stress‐associated gastric mucosal damage via its anti‐inflammatory, antioxidant and anti‐apoptotic effects.^6^, ^7^, ^8^ A new technology based on electrolysis of water has been suggested for clinical amelioration of several pathologies. Electrolysed reduced water (ERW), rich in H_2_, generated at the cathode during water electrolysis, has a high pH, low dissolved oxygen and an extremely negative redox potential (ORP).^9^ Moreover, in our recent study, we have demonstrated that human histiocytic lymphoma cells line U937 cultured in an ERW‐medium could alleviate H_2_O_2_‐induced cytotoxicity of cells through the modulation of cellular redox state.^10^ Gastroesophageal reflux disease (GERD) is a clinical condition in which the reflux of gastric contents into the oesophagus induces complications and complex symptoms, impairing quality of life.^11^, ^12^ Even if the gastric mucosal acts as a protective barrier, pathogens and ingested materials can induce an unbalance of the redox cell state and GI inflammatory responses.^13^ In fact, several studies have highlighted that oxidative stress is involved in the development and progression of several GI disorders such as GERD, enteritis, gastritis, peptic ulcer, GI cancers and colitis.^14^, ^15^ ROS are produced within the GI tract, but their involvement in pathophysiology of GERD have not been well investigated.^13^, ^16^ The production of ROS in cell systems is attributable to the activity of many enzymes such as peroxidases, xanthine oxidase, NADPH oxidase, NADPH oxidase isoforms, glucose oxidase, lipoxygenases, myeloperoxidase and cyclooxygenases.^13^, ^17^ Proton pump inhibitors (PPIs) have been universally accepted as a first‐line therapy for management of GERD and are among the most commonly prescribed medicines for gastroesophageal reflux and peptic ulcer disease.^18^ PPIs block acid production irreversibly inhibiting H^+^/K^+^ adenosine triphosphatase in gastric parietal cell.^19^ Omeprazole, the first drug in this class, was introduced in 1989 and was followed by lansoprazole (1995), pantoprazole (2000), esomeprazole (2001) and dexlansoprazole (2009). Current guidelines recommend empiric therapy with PPIs for patients suspected of having GERD.^11^ Despite their efficacy, several studies have shown that a significant proportion of GERD patients are either partial or non‐responders to PPI therapy. In a recent article in JAMA Internal Medicine, some researchers report data on the negative effects of the often overuse of PPIs, widely used in the United States (as well as in Italy, as pointed out by OsMed data).^20^ A series of systematic reviews have brought further evidence to support the thesis that PPIs are overprescribed and are associated with a number of adverse effects. Numerous observational studies have documented probable causal links with the use of PPIs and adverse reactions, including acute and chronic kidney disease, fractures, hypomagnesaemia, bacterial infections and cardiovascular risk.^21^, ^22^, ^23^, ^24^, ^25^ Thus, GERD reduces the quality of life and significantly affects the health care system.^26^ For these reasons, the aim of this study was to assess the efficacy of H_2_‐rich water, called ERW, in modulating the symptoms and systemic oxidative stress associated with GERD. We hypothesize that the ERW could be considered as supplementary treatment for GERD, because it could reduce the heartburn and regurgitation in turn enhancing the well‐being of the patients. Thus, the goal of our study was to investigate whether ERW reduces the plasmatic level of oxidative stress in ex vivo peripheral blood mononuclear cells (PBMCs) of GERD patients, relating to scores GERD, as total score (TS), heartburn score (HS) and regurgitation score (RS). Altogether 84 patients reporting moderate to severe heartburn and regurgitation symptoms underwent control treatment (PPI + tap water) or experimental treatment (PPI + ERW) for 3 months. Our findings demonstrated that experimental treatment improves the oxidative balance through a reduction in typical GERD symptoms such as heartburn and regurgitation compared to control treatment.

## 2 MATERIALS AND METHODS

### 2.1 Electrolysed reduced water

Electrolysed reduced water was prepared as described previously using the medical device *Alka vitha*.^10^ The apparatus for the electrolysis of water consists of an active carbon filter (0.2 μm) for water purification and a Pt‐coated Ti electrode for water electrolysis. Furthermore, the apparatus has a pH control system (pH 8.10‐11.60) and *Eh* values from −200 to −800 mV. The *Eh* represents the redox potential of an aqueous solution, and it is a measure of the reductive power ability of dissolved molecular hydrogen (H_2_).

### 2.2 Patients

We enrolled a group of drug‐naïve patients with a diagnosis of GERD. The diagnosis was carried out in accordance with the guidelines for GERD.^27^ The study was conducted in compliance with the “ethical principles for medical research involving human subjects” of the Helsinki Declaration. The local ethics committee has revised and finally approved this study **(**trial registration: number CE; 992 of 2015/07/07). The individuals were patients of the “Sant.ma Annunziata” Hospital of Chieti between September 2015 and March 2016. The study follow‐up ended on June 2016. The study included adults (age ≥ 18 years) who had a diagnosis of GERD, with a history of frequent episodes of GERD‐related symptoms (regurgitation, heartburn, retrosternal pain) for more than a month prior to the study screening. Patients were excluded from the study if they had experienced one of the following conditions within the previous 3 months: acute infections, vascular access thrombosis, acute myocardial infarction, stroke, diabetes, clinically relevant bleedings, major surgical procedures, blood transfusions, systemic inflammatory of metabolic diseases, active malignancies, smoking habit and participation in other experimental clinical studies. Moreover, patients were also excluded if they suffered from any type of GI disorders, gastroduodenal ulcers, Barrett's oesophagus, use of concomitant therapy, as well as alcohol or drug abuse. Patients with a BMI of <20 and >33 kg/m^2^, as well as unusual dietary habits (eg vegetarians), were also excluded. The participants of study were subjected to a blood sample and submitted to GERD‐Health Related Quality of Life Questionnaire (GERD‐HRQL), to define successful response both clinically and systemically to the 3‐month dose of PPI or ERW + PPI. C‐reactive protein (CRP) was measured as a non‐specific marker for inflammation. All the patients underwent 2 monitoring visits, at baseline (*t*_0_) and after 3 months (*t*_1_).

### 2.3 GERD‐Health Related Quality of Life Questionnaire (GERD‐HRQL)

The Gastroesophageal Reflux Disease‐Health Related Quality of Life (GERD‐HRQL) instrument is a self‐administered questionnaire introduced to provide a quantitative method of measuring frequency and severity of GI symptoms in gastroesophageal reflux disease (GERD). The purpose of GERD‐HRQL was to measure symptomatic change as a result of medical or surgical treatment of GERD. The GERD‐HRQL instrument is practical and generally administered by simply handing it to the patient during a screening visit.^28^ The questionnaire measuring 16 items (6 related to heartburn, 2 to dysphagia, 6 to regurgitation, 1 to the impact of medication on daily life and 1 on the satisfaction level) on the VAS scale from 0 (no symptoms) to 5 (worst symptoms). The results are expressed as TS, heartburn score (HS) and RS. TS was calculated by summing the individual scores to questions 1‐15 with scores ranging from 0 (no symptoms) to 75 (worst symptoms). HS was calculated by summing the individual scores to questions 1‐6 with scores ranging from 0 (no heartburn symptoms) to 30 (worst heartburn symptoms). RS was calculated by summing the individual scores to questions 10‐15 with scores ranging from 0 (no regurgitation symptoms) to 30 (worst regurgitation symptoms). Satisfaction level‐related quality of life was measured considering the responses at treatment experience assessing in satisfied, neutral and not satisfied.

### 2.4 Isolation of human peripheral blood mononuclear cells

Blood samples for laboratory screening were collected at t_0_ (before administration of ERW or tap water + PPI) and t_1_ (at study end‐point) in 4‐mL endotoxin‐free Heparin tubes (Vacutainer; Becton Dickinson, NJ, USA). Venipuncture was performed in the morning (08.00‐10.00 am.) after an overnight fast and before breakfast. Tubes were kept at room temperature and transported to the laboratory for processing within 1 hour of collection. PBMCs were isolated by density‐gradient centrifugation through Ficoll‐Hypaque (Pharmacia) as described previously.^29^ Cell viability in each culture was assessed by Trypan blue die exclusion. All solutions were prepared using pyrogen‐free water and sterile polypropylene plastic‐ware and were free of detectable LPS (<0.1 EU/mL), as determined by the Limulus amoebocyte lysate assay (sensitivity limit 12 pg/mL; Associates of Cape Cod, MA, USA). All reagents used were tested before use for mycoplasma contamination (minimum detection level 0.1 μg/mL) (Whittaker Bioproducts, Walkersville, MD, USA) and found negative. The same batches of serum and medium were used in all experiments. After 24 hours incubation, samples were centrifuged at 400 *g* for 10 minutes at room temperature and supernatants were collected and stored at −80°C until assay. The PBMCs yield per ml of blood was approximately 1 × 10^6^ cells. The plasma was obtained by blood centrifugation as described previously and was kept frozen at −20°C.^30^

### 2.5 Assessment of oxidative stress

Plasma was tested for total oxidant capacity and antioxidant potential using a derivatives reactive oxygen metabolites (d‐ROMs) and a biological antioxidant potential (BAP) test kit (Diacron International s.r.l., Grosseto, Italy), respectively.

#### 2.5.1 d‐ROMs test

The test is based on the concept that the amount of organic hydroperoxides present in serum is related to the free radicals from which they are formed. Serum sample is dissolved in an acidic buffer (pH 4.8). The d‐ROMs test is based on the ability of a plasma sample to oxidize the chromogen substrate (N‐N‐diethylparaphenilendiamine) to its radical cation; the reaction is monitored photometrically at 37°C at 505 nm, and the results are expressed as Carratelli Units (CARR U, ΔAbs5050 nm/min), where 1 U‐CARR. corresponds to 0.8 mg/L H_2_O_2_. The normal values of the test are between 250 and 300 U‐CARR. (Carratelli Units Values) outside this range are considered indicative of an alteration in the equilibrium between pro‐oxidant and antioxidant capability of patients. Values >300 U‐CARR. indicate a condition of oxidative stress.

#### 2.5.2 BAP assay

Through this test, the components of the antioxidant plasma barrier were measured directly by the active scavengers. The BAP test was performed according to the manufacturer's instructions (Diacron). A chromogen reagent containing trivalent iron was added to a plasma sample. BAP assay is based on the ability of a plasma sample to reduce Fe^3+^ to its colourless ferrous derivative (Fe ^2^). The reaction is monitored by photometric reading at 37°C at 505 nm, and the results are expressed in μEq/L of reduced iron using vitamin C as a standard. The optimal value of a BAP test is >2200 μEq/L. Values lower than 2.200 μEq/L indicate a reduced “biological potential” and hence a decreased effectiveness of the antioxidant plasma barrier, according to an arbitrary scale of severity.

#### 2.5.3 Nitro blue tetrazolium (NBT) assay

The production of intracellular superoxide anion was performed using nitro blue tetrazolium (NBT) (Sigma‐Aldrich SRL, Milano, Italy, Catalog No: N6639) as described previously.^31^ After PBMC extraction, cells were incubated with NBT (0.1 mg/mL) in culture medium for 3 hours at 37°C; and were further washed 3 times with methanol. The amount of NBT‐formazan produced is an index of O_2_^−^ intracellular level. After the solubilization of crystals in 200 mL of KOH 2M/DMSO solution, the quantization was determined spectrophotometrically (Spec‐traMaxH 190; Molecular Devices) at 630 nm. The results were expressed as nmol/mL of O_2_^−^ released.

#### 2.5.4 Griess assay

The assay was carried out as described previously.^32^ Two ×10^6^ cells were seeded in 6 wells/plates, and nitrite was measured in culture supernatants as an indicator of the nitric oxide production. Aliquots of the culture supernatant were mixed with an equal volume of the Griess reagent (Sigma‐Aldrich, USA; Catalog No: G4410) and absorbance was determined at 540 nm using a microplate reader. Sodium nitrite, at concentrations of 0 to 100 μM, was used as a standard to assess nitrite concentrations.

### 2.6 Measurement of CRP

The amount of circulating CRP levels was assayed using specific ELISA development systems (Diagnostics Biochem Canada Inc, Neptune Crescent, London, ON, Canada, Catalog No: CAN‐CRP‐4360). The experiments were performed in triplicate according to the manufacturer's instructions. CRP values are expressed as mg/L. The CRP assay sensitivity was <10 ng/mL. The intra‐ and inter‐assay reproducibility was >90%. Triplicate values that differed from the mean by more than 10% were considered suspect and were repeated.

### 2.7 Measurement of malondialdehyde (MDA)

MDA levels were assayed using specific ELISA development systems (Elabscience; Catalog No: E‐EL‐0060). Plates were scanned using a specialized charge coupled device cooled tool. The integrated density values of the spots of known standards were used to generate a standard curve. Density values for unknown samples were determined using the standard curve for each patient to calculate the real values in pg/mL. All steps were performed in triplicate and at room temperature. The MDA assay sensitivity was <18.75 ng/mL. The intra‐ and inter‐assay reproducibility was >90%. Triplicate values that differed from the mean by more than 10% were considered suspect and were repeated.

### 2.8 Statistical analysis

The quantitative variables were summarized as mean and standard deviation (SD) or median and interquartile range (IQR), according to their distribution. Qualitative variables were summarized as frequency and percentage. A Shapiro‐Wilk's test was performed to evaluate the departures from normality distribution for each variable. An analysis of variance (ANOVA) for repeated measures was performed to evaluate the effect of time (baseline vs post‐therapy), group (PPI vs PPI + ERW) and their interaction on laboratory parameters. Chi‐square test was performed to evaluate differences in distribution of d‐ROMs test and BAP test between groups when analysed as categorical data. A Friedman's test was performed to evaluate the differences in GERD total scores, heartburn score and regurgitation score from baseline to post‐therapy. Mann‐Whitney *U*‐test was performed to evaluate differences in score relative variation between groups. Spearman's correlation coefficient (*Ρ*) was performed to evaluate the correlation among laboratory parameters and scores. The false discovery rate correction (FDR) was used to control the family‐wise type I error rate and an FDR‐adjusted *P*‐value < .05 was determined to be statistically significant. Statistical analysis was performed using IBM^®^ SPSS Statistics v 20.0 software (SPSS Inc, Chicago, IL, USA).

## 3 RESULTS

### 3.1 Patients

**Figure 1:**
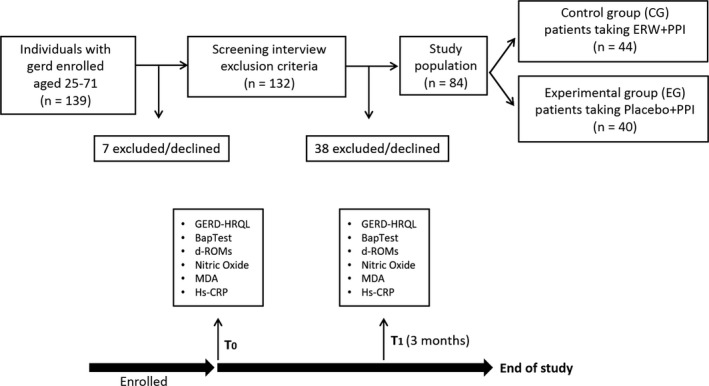
Flow diagram of patient selection

As reported in Figure 1, 139 patients took part in the study, 7 of these withdrew while 38 were excluded after the screening interview. In the end, 84 consecutive individuals were included in the study. After giving their written informed consent, the patients were assigned to the control treatment (PPI + tap water) or to the experimental treatment (PPI + ERW) for 3 months. According to the protocol, on a daily basis, the participants drank 1.500 mL of ERW containing dissolved H_2_ or tap water. All patients included into the experimental treatment received the medical device for the time set for the study. Firstly, all patients received a shock treatment of pantoprazole, 40 mg⁄d, orally for 4 weeks and then 20 mg⁄d for 8 weeks. Pantoprazole was taken 30 minutes before breakfast for a period of 3 months. Of the 84 patients with GERD who were enrolled in this survey, 44 patients were female and 40 patients were male. The mean age of the patients was 51.95 ± 10.90 years, ranging from 23 to 71 years of age. The patients were randomized into PPI (control group‐CG‐) and PPI + ERW (experimental group‐EG‐) groups. Of the 40 patients included in the control group (CG), the mean age as 52.3 ± 10.7 years, 18 patients were male (45%) and 22 patients were female (55%). Of the 44 individuals included in the EG, with mean age of 51.6 ± 11.1 years, 22 patients were male (50%) and 22 patients were female (50%). Statistical analysis showed no statistical differences between the 2 groups regarding age, gender and BMI.

### 3.2 Quality of life outcome

The typical symptoms of GERD include heartburn and regurgitation, occurring both during the night, frequently waking the patient up from sleep, and during the day, frequently associated with meals which have a great impact on a patients' quality of life.^33^ Table 1 shows the difference of the frequency of GERD presentations, before and after treatment among all the patients. As the table shows, the frequency of presentations decreased in both CG and EG groups after treatment. Baseline GERD total scores were 63.0 (53.8‐71.0) and 56.5 (47.3‐64.8) in the CG and EG groups, respectively (*P* < .05). Post‐treatment results were 38.0 (30.0‐46.0) and 27.5 (19.5‐37.8) in the CG and EG groups, respectively (*P* < .001), with a relative variation of 0.4 and 0.5, respectively (*P* = .013). Baseline HS and RS were, respectively, 25.0 (20.3‐27.0) and 25.0 (21.3‐27.0) for the CG and 23.5(20.0‐26.0) and 25.0 (21.3‐28.0) for the EG groups. Post‐treatment results were, respectively, 15.0 (12.0‐19.0) and 15.5 (12.0‐18.0) for the CG and 7.0 (4.0‐12.0) and 7.5 (4.0‐11.0) for the EG. The effect of time was significant for all considered scales (*P* < .001). Relative variation of HS and RS were, respectively, −0.4 for the CG, −0.7 for the EG group (both *P* < .001). At 3 months' follow‐up, the median GERD‐HRQL scores improved significantly after treatment both in CG and in EG groups (38.0 CG vs 27.5 EG), but the statistical analysis revealed that in the patients that associate with the intake of PPI also ERW there is a better significance in relation to HS and RS parameters (*P* < .001). In summary, treatment with ERW + PPI, for 3 months, gave significantly better symptom control than PPI treatment. Finally, in our study, 75% of the patients studied report a good satisfaction level after ERW treatment. Taken together the results showed that there was significant increase in quality of life at 3 months after supplementation with ERW when compared to baseline (*P* < .005).

**Table 1:** Differences in scores variation expressed as median and interquartile range

	Baseline	Post‐treatment	Relative variation	*P*‐value^a^	*P*‐value^b^
TS Item GERD					
CG	63.0 (53.8; 71.0)	38.0 (30.0; 46.0)	−0.4 (−0.5; −0.2)	**<.001**	**.013**
EG	56.5 (47.3; 64.8)	27.5 (19.5; 37.8)	−0.5 (−0.7; −0.4)		
HS					
CG	25.0 (20.3; 27.0)	15.0 (12.0; 19.0)	−0.4 (−0.5; −0.2)	**<.001**	**<.001**
EG	23.5 (20.0; 26.0)	7.0 (4.0; 12.0)	−0.7 (−0.9; −0.5)		
RS					
CG	25.0 (21.3; 27.0)	15.5 (12.0; 18.0)	−0.4 (−0.5; −0.3)	**<.001**	**<.001**
EG	25.0 (21.3; 28.0)	7.5 (4.0; 11.0)	−0.7 (−0.8; −0.5)		

CG, control group (PPI + TAP water); EG, experimental group (PPI + ERW); TS, total score; HS, heartburn score; RS, regurgitation score; ERW, electrolysed reduced water; PPI, proton pump inhibitors.

a Effect of time assessed by Friedman's test.

b Differences between PPI and PPI + ERW therapy group assessed by Mann‐Whitney *U*‐test.

Bolded *P*‐values are significant after FDR correction.

### 3.3 Effect of ERW on oxidative stress in GERD patients

Laboratory parameters trends in the CG and EG groups during follow‐up are reported in Table 2. Several studies have been highlighted that inflammatory cytokines and oxidative stress are involved in the development and progression of GERD.^34^ Our results confirmed that patients affected by GERD presented higher levels of systemic nitrosative and oxidative stress at baseline. On recruitment, the mean values of nitric oxide, MDA and O_2_^−^ were 61.75 ± 24.90 nmol/mL/10^6^ cells, 193.45 ± 121.20 pg/mL and 89.66 ± 24.60 nmol/mL, respectively. Moreover, the analysis of the balance between ROS and antioxidant barrier demonstrated that the values of d‐ROMs and BAP test in GERD patients at baseline were 394.05 ± 110.65 U‐CARR and 847.15 ± 443.05 μEq/L, respectively. Our data are consistent with Wetscher et al^35^, who observed that free radicals/active oxygen species are involved in the pathogenesis of reflux oesophagitis. After treatment, the balance between ROS and antioxidant barrier were generally found to have progressively returned to normal range. Indeed, the follow‐up visit at 3 months after treatment (t_1_) revealed an average reduction in the value of the d‐ROMs test and an average increase in the value of the BAP test. ANOVA test for repeated measures indicated a significant difference for nitric oxide level (*P* = .025) and BAP test (*P* < .001) between 2 groups. Nitric oxide levels were significantly decreased in EG vs. CG (57.2 ± 12.29 vs 41.1 ± 14.9; *P*‐value < .001). These data are supported by the remarkable increase in the antioxidant barrier in EG patients compared to controls (798.1 ± 339.3 vs 1796.7 ± 467.2; *P*‐value < .001). Significant effect of period (*P* < .001) was found for all laboratory parameters. Interaction group × period was significant for all parameters (*P* < .001) except for CRP. These values indicated a positive modulation of the pro‐oxidant/antioxidant balance with a reduction in oxidative damage in GERD patients. In addition, we analysed the severity of oxidative stress and of antioxidant barrier impairment (Table 3). On recruitment, about the same percentage of the patients belonging to CG and EG exhibited highly oxidative stress (>500 U‐CARR). Moreover, at the t_0_, 92.5% of patients belonging to CG and 88.6% of patients in EG had a very strong reduction in the antioxidant barrier (BAP test value < 1400). After 3 months of treatment (t_1_), no changes in antioxidant barrier were observed in the CG. Notably, in the EG, 23.3% of patients fall within the optimum range of antioxidant barrier and the 53.5% have an optimal value of plasmatic oxidative stress.

**Table 2:** ANOVA for repeated measures performed to evaluate pre‐ and post‐therapy parameters between PPI + TAP water (CG) and PPI + ERW (EG) therapy group

	Baseline	Post‐treatment	*P*‐value*	*P*‐value**	*P*‐value***
CRP (mg/L)					
CG	2.3 ± 2.2	1.6 ± 1.6	**<.001**	.839	.455
EG	2.2 ± 1.6	1.4 ± 1.1			
NO (nmol/mL/10^6^ cells)					
CG	59.3 ± 13.6	57.2 ± 12.9	**<.001**	**.025**	**<.001**
EG	64.2 ± 11.3	41.1 ± 14.9			
MDA(pg/mL)					
CG	190.3 ± 106.8	203.0 ± 112.0	**.001**	.084	**<.001**
EG	196.6 ± 135.4	117.9 ± 91.6			
d‐ROMs test (U‐CARR)					
CG	385.1 ± 86.4	380.9 ± 71.6	**<.001**	.062	**<.001**
EG	403.0 ± 134.9	292.2 ± 89.2			
Biological antioxidant potential test (μEq/L)					
CG	839.2 ± 441.2	798.1 ± 339.3	**<.001**	**<.001**	**<.001**
EG	855.1 ± 444.9	1796.7 ± 467.2			
O_2_^−^ (nmol/mL)					
CG	83.53 ± 21.00	78.1 ± 14.3	**<.001**	.218	**<.001**
EG	95.8 ± 28.2	57.1 ± 21.2			

CG, control group; EG, experimental group; CRP, C‐reactive protein; NO, nitric oxide; MDA, malondialdehyde; O_2_^−^, superoxide anion; d‐ROMs, derivatives reactive oxygen metabolites; ERW, electrolysed reduced water; PPI, proton pump inhibitors.

Bolded *P*‐values are significant after FDR correction.

Probability that effect on the addressed variable is influenced by: *period. For each variable, the differences have been tested between the means of each period of the 2 groups (CG and EG); **groups. For each variable, the differences have been tested between the means of PPI group in 2 time (Baseline and post‐treatment) and the means of the EG group in 2 time; ***probability that the effects of period is greater in one distinct group (interaction period × group).

**Table 3:** Difference in d‐ROMs and biological antioxidant potential (BAP) test measurements between CG (PPI + Tap Water) and EG (PPI + ERW) group

	Baseline			Post‐treatment		
	CG n (%)	EG n (%)	χ^2^ *P*‐value	CG n (%)	EG n (%)	χ^2^ *P*‐value
d‐ROMs test (*U*‐CARR)						
<300	8 (20.0)	9 (20.5)	.290	4 (10.0)	23 (53.5)	**<.001**
300‐320	3 (7.5)	3 (6.8)		3 (7.5)	3 (7.0)	
321‐340	2 (5.0)	5 (11.4)		5 (12.59	5 (11.6)	
341‐400	12 (30.0)	6 (13.6)		13 (32.5)	9 (20.9)	
401‐500	12 (3.0)	12 (27.3)		14 (35.5)	1 (2.3)	
>500	3 (7.5)	9 (20.5)		1 (2.5)	2 (4.7)	
BAP test (μEq/L)						
≥2200	0	0	.393	0	10 (23.3)	**<.001**
2200‐2001	0	0		0	4 (9.3)	
2000‐1801	3 (7.5)	3 (6.8)		0	9 (20.9)	
1800‐1601	0	0		1 (2.5)	8 (18.6)	
1600‐1401	0	2 (4.5)		2 (5.0)	3 (7.0)	
≤1400	37 (92.5)	39 (88.6)		37 (92.5)	9 (20.9)	

CG, control group; EG, experimental group; d‐ROMs, derivatives reactive oxygen metabolites; ERW, electrolysed reduced water; PPI, proton pump inhibitors.

χ^2^ *P*‐value = Chi‐squared test. *p* value < 0,05 are considerated statistically significant.

### 3.4 Correlation between laboratory parameters and GERD

Spearman's correlation coefficient was used to evaluate the link among scores and laboratory parameters. TS relative variations correlated with laboratory parameters relative variations, except for BAP test, as shown in Table 4. HS and RS relative variations were significantly correlated with laboratory parameters variation, except for PCR. BAP was significantly associated with HS and RS reduction (ρ = −.439 and −.505, respectively).

**Table 4:** Spearman's correlation coefficient assessed to evaluate correlation among scores relatives variation and laboratory parameters relative variation

	TS	HS	RS
CRP (mg/L)			
ρ	.245	.092	.088
*P*‐value	**.025**	.407	.424
NO (μmol/L/10^6^ cells)			
ρ	.341	.384	.423
*P*‐value	**.001**	**<.001**	**<.001**
MDA(pg/mL)			
ρ	.469	.363	.344
*P*‐value	**<.001**	**.001**	**.001**
d‐ROMs test (U‐CARR)			
ρ	.414	.371	.310
*P*‐value	**<.001**	**.001**	**.004**
BAP test (μEq/L)			
ρ	−.170	−.439	−.505
*P*‐value	.123	**<.001**	**<.001**
O_2_^−^ (nmol/mL)			
ρ	.398	.350	.294
*P*‐value	**<.001**	**.001**	**.007**

CRP, C‐reactive protein; NO, nitric oxide; MDA, malondialdehyde; O_2_^−^, superoxide anion; TS, total score; HS, heartburn score; RS, regurgitation score; BAP, biological antioxidant potential.

Bolded *P*‐values are significant after FDR correction.

## 4 DISCUSSION

GERD is characterized by a number of symptoms, the 2 most common being frequent heartburn and regurgitation.^11^ For these patients, proton pump inhibitors (PPIs) have been widely adopted as first‐line therapy management of GERD and represent the gold standard therapy. PPIs act by blocking the proton pump of the gastric parietal cells, thus inhibiting a large percentage of acid secretion over 24 hours. Nowadays, there is no evidence that PPIs therapy can prevent the onset of erosion and its progression to pathological lesion.^24^, ^36^ The oesophageal mucosa has the intrinsic capacity to resist pathogenic damage, which makes it suitable to self‐protection and regeneration. This intrinsic capacity of regeneration could be the basis of the metaplasia. From the point of view of cell growth, unfortunately oesophageal epithelium is less studied. There are at least 3 different levels of intrinsic defence in the oesophageal mucosa. The first level is pre‐epithelial and is represented by the surfactant, a liquid film deposited on the mucous membrane, which because of its visco‐elastic properties, mechanically protects the epithelium and avoids that the lytic substances come into contact with it. The second level is intra‐epithelial and it is represented by the layer of epithelial cells, which through their relative tight junctions prevent the penetration of H^+^ ions. The third level is post‐epithelial and is represented by the regulatory mechanism of cell tropism.^37^, ^38^ When there is a correct tissue blood flow, the tissue oxygenation and the process of neutralization of free radicals play a role in the maintenance of an effective tissue homeostasis.^39^ In patients with GERD, an adequate blood supply ensures hyperaemia, which leads to infiltration of neutrophils and eosinophils cells in the oesophageal mucosa, causing cell necrosis. In recent years, oxidative stress has been postulated to be an important factor in the pathogenesis and development of lifestyle‐related disease, such as gastroesophageal reflux.^21^ It is strongly agreed that ROS and reactive nitrogen species (RNS) are generated during inflammation and are considered to contribute to flogosis leading to carcinogenesis.^40^ In fact, chronic inflammation during GERD is an important risk factor of Barrett's oesophagus (BE) and oesophageal carcinogenesis.^3^ The goal of reflux treatment is not necessarily, the complete absence of symptoms, the healing of major oesophageal lesion and the prevention of complications.^41^ ROS and RNS can induce the formation of a variety of molecule markers of oxidative and nitrosative damage, such as the production of superoxide anion (O_2_^−^) and nitric oxide (NO). In the condition of oxidative stress, nitric oxide was produced through the activation of inducible isoform iNOS with formation to elevate concentration of nitric oxide and thus of peroxynitrite (ONOO^−^). As nitric oxide is a main signalling molecule in cells, its overproduction may lead to pathological effects in several organ systems.^29^, ^42^, ^43^ Wide quantities of nitric oxide were found in human gastro‐junction, and it can diffuse epithelial mucosa and contribute to the increase in the GERD pathological condition. ROS levels have been reported to be increased in oesophagitis compared to healthy controls in both patients and murine models and are hypothesized to mediate mucosal damage and drive disease progression.^44^, ^45^ Administration of many antioxidants have been shown to prevent mucosal damage in models of oesophagitis suggesting that antioxidant treatment should be considered as a therapy in the treatment of oesophagitis.^33^, ^45^ Alternative treatments are commonly used for various disorders and are often taken on‐demand. There is an increasing use of complementary and alternative medicine that, in contrast to drugs, is believed to be harmless.^41^, ^46^ Medical research has shown in some studies that the H_2_ molecule can have an antioxidant and cytoprotective role in several diseases. With the recent progress of H_2_ science, considering the report that H_2_ gas could reduce cytotoxic oxygen radicals, therapeutic application of H_2_ has become a clinical challenge. Recently, several studies have revealed that ERW, enriched of H_2,_ has a unique biological capacity to act as an antioxidant and anti‐inflammatory substance.^47^, ^48^ The consumption of ERW has also been shown to exhibit scavenging activity.^49^ Kashiwagi et al^50^ showed in a recent study how ERW supplies a DNA protection from free radicals damage. Many have reported in the last few years that GERD is a complex inflammatory disease characterized by the recruitment of factors related to inflammation such as chemokines, cytokines, oxidative stress, growth factors and inflammatory cells.^34^, ^51^ Our hypothesis is that H_2_, being an extremely volatile and permeable gas, crosses the plasma membrane with the ability to react with toxic radicals neutralizing them. In this new original study, we recruited 84 patients with GERD, divided into 2 groups, control group (CG) and an EG. The statistical analysis shows that in the 2 groups studied, PPI therapy improves GERD‐related symptomatology (Table 1). Supplementation of standard therapy with ERW gave significantly better symptom control than PPI treatment. In GERD patients, it was noted how problems linked to drinking, eating, pain, sleeping, compromises life's quality. In actual fact, it is known that people with this disorder have a lower quality of life than those without GERD. Our results demonstrated that the reduction in clinical symptoms such as heartburn and regurgitation leads to a statistical improvement of the quality of life, as demonstrated by the analysis of satisfaction levels, at 3 months after supplementation with ERW when compared to baseline. In addition, we observed a higher significant difference between the 2 groups at t_1_, not only in reducing of clinical symptoms, but also an elevated reduction in MDA level, a clear index of a considerable decrease in lipid peroxidation (Table 2). These results also supported a marked reduction in nitric oxide production which was statistically significative in EG respect to the CG. The assessment of oxidative stress is an important but technically challenging procedure in medical and biological research. Jiménez et al^52^ reported that a decrease in antioxidant activity leading to increased mucosal levels of superoxide anion and peroxynitrite radicals may contribute to the development of oesophageal damage and Barrett's oesophagus in patients with GERD. Accordingly, our results demonstrated that GERD is associated with a clear alteration of cellular redox state, which is characterized by a profound increase in O_2_^−^ production, an increase in nitric oxide and MDA levels (Table 2). To confirm these data, we evaluated derivate reactive oxygen metabolites (d‐ROMs) and BAP in GERD patients. We noted that after treatment, reduction in oxidative stress in plasma is present in both groups, but notably, in the EG, 23.3% of patients return to the optimum range of antioxidant barrier (<2200 μEq/L), while the 92.5% of CG patients have a strongly compromised antioxidant barrier (Table 3). Furthermore, increased BAP test was significantly associated with HS and RS reduction (ρ = −.439 and −.505, Table 4). Thus, the combination of ERW and PPI was shown to be effective in decreasing the scores of GERD and in decreasing oxidative injury‐mediated by nitric oxide and O_2_^−^ in GERD patients. These findings signify that ERW supplementation and subsequent ROS reduction together could be used to improve oesophageal damage. These new results, along with our previous results, are in accordance with in vitro research experiments by Hamasaki and his group, which made evident that ERW neutralizes ROS, in a very similar process to the action of SOD and CAT enzymes.^53^ As GERD is characterized by excessive production of free radicals in the GI system exceeding the endogenous system's capability to neutralize and eliminate them, we conclude that oxidative stress should be modulated to maintain cellular homeostasis. Therefore, balanced redox status through the optimal modulation of oxidative stress or homeostasis could be essential in considering antioxidant therapy for the prevention of inflammation‐based GI disorder. Our results demonstrate that in GERD patients, combination treatment with PPI and ERW improves the cellular redox state leading to the improvement of the quality of life as demonstrated by the correlation analysis between laboratory parameters and GERD. H_2_ easily penetrates cells by diffusion and, without disturbing metabolic redox reactions, reduces oxidative stress because of its ability to react with strong oxidants. Our hypothesis is that H_2_, acting as a scavenger against the ${}^{\cdot }O{{}_{2}}^{-}$ and the ${}^{\cdot }\text{OH}$, neutralizes the toxicity induced by these radical species with consequent reduction in the formation of ONOO^−^. This leads to a significant lowering in the oxidative systemic damage, which results in a minor infiltration of the inflammatory cells thus in lowering the local hyperaemia and returning the redox cell balance. The increase in the plasma antioxidant barrier and the reduction in free radicals lead to a reduction in the flogosis, decreasing patient symptomatology and improving quality of life. Moreover, GERD is linked to exclusive use of therapy with PPIs as well as a correct lifestyle, and this entails considerable expenditure on health care system. This treatment, for a large number of patients, is not efficient (PPIs non‐responders) and one must not exclude the adverse effects of its prolonged use. Clinicians must be aware of the potential risks and ensure the supervision of the prescriptions of PPIs use must be tailored, using a personalized therapy. Our study is innovative and of great social impact because it highlights that in GERD patients, using a combination regimen with PPI and ERW, rich in molecular hydrogen (H_2_), as a therapy, can provide systemic changes such as a reduction in heartburn and regurgitation symptoms as well as a major improvement of the quality of life. The future perspectives may be based on the hypothesis of using ERW as neoadjuvant/coadjuvant therapy with PPI at decreasing doses for the treatment of GERD.

## CONFLICTS OF INTEREST

We state that there is no conflict of interest and declare that we have no financial and personal relationship with other people or organizations that could influence this work.
